# Post-coital posterior fornix perforation with vaginal evisceration

**DOI:** 10.1186/s12905-014-0141-6

**Published:** 2014-11-25

**Authors:** Alex Ernest, Mtui Emmanuel, Knapp Gregory

**Affiliations:** Department of Surgery and Maternal health, University of Dodoma, College of Health Sciences, Dodoma, Tanzania; Department of Surgery, Halifax, Dalhousie University, Faculty of Medicine, Halifax, Canada

**Keywords:** Post coital, Posterior fornix perforation, Vaginal evisceration

## Abstract

**Background:**

Cases of post-coital posterior fornix perforation with vaginal eviscerations are infrequently reported in literature and uncommon cause for laparotomy.

**Case presentation:**

We report the case of 28 year old nulliparous woman presented to the hospital with per vaginal bleeding and evisceration following penile-vaginal sexual intercourse.

**Conclusion:**

High degree of suspicion in these cases is important especially in sexually active women as delay in management often results in life threatening blood loss, peritonitis and intestinal obstruction. Physicians should be aware that initial patient history may be inaccurate or misleading if taken in the presence of family or partner given the sensitive nature of the injury.

## Background

It is not uncommon to encounter non-obstetric injuries of the genitourinary system. Several cases of posterior vaginal fornix injury have been reported in the literature. However, there is a paucity of information on the presentation and management of post-coital vaginal perforation. We report a case of post-coital posterior fornix perforation with hollow organ evisceration requiring laparotomy.

## Case presentation

A 28-year-old nulliparous woman, presented at Iringa Region Hospital with a one day history of abdominal pain and bright red blood per vagina. In the presence of her grandmother she denied any history of coitus or vaginal instrumentation prior to the onset of symptoms. However, when interviewed alone she reported engaging in penile-vaginal intercourse, without foreign-body instrumentation, several hours prior to onset of symptoms.

The patient described a sudden onset of generalized, lower abdominal pain. Initially the abdominal pain was sharp in quality but gradually became dull with radiation to the back and to the tip of the shoulder. This was accompanied shortly thereafter with frank vaginal bleeding and protrusion of mass per vagina notably during defecation and micturition. Initially the mass was reducible but later it become irreducible. This was associated with significant nausea, bilious emesis, pre-syncope and palpitations. She denied any prodrome of fever, diarrhea or rectal bleeding. Her past medical history was unremarkable.

On presentation to hospital she was in obvious distress. She was clinically hypovolemic with sunken eyes and dry mucosal membranes with a mildly elevated heart rate at 80 beats per minute and a blood pressure of 100/60 mm/Hg. Her body temperature was 36.9°C. The abdomen was rigid with generalized involuntary guarding, rebound tenderness and hyperactive bowel sounds.

**Figure 1:**
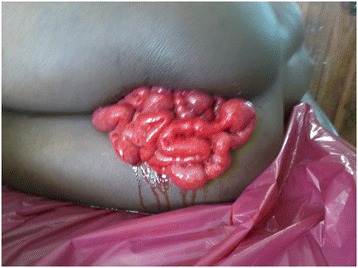
**Patient presentation at admission**- **bowel evisceration with bright red blood oozing.**

On examination of the perineum, non-reducible intra-abdominal contents were clearly visualized protruding from the vaginal os (Figure 1). A digital rectal exam was performed, which was unremarkable, with normal sphincter tone and no obvious injury. A speculum examination was not technically feasible due to the edematous, incarcerated bowel.

**Figure 2:**
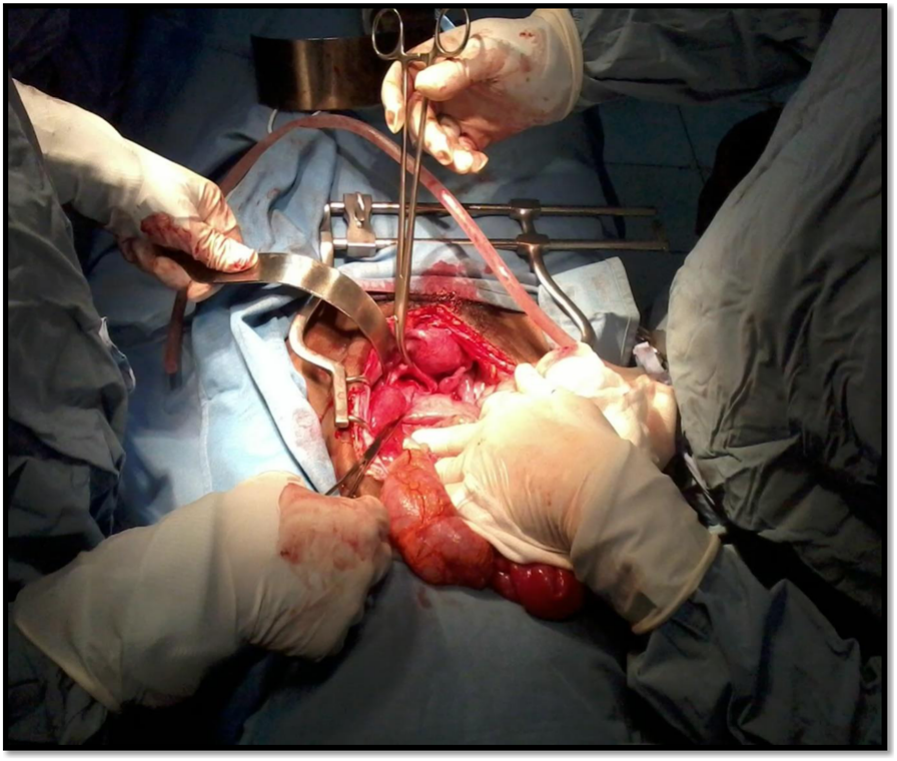
**Intraoperative findings:** **a full length tear of the posterior fornix with an intact uterus.**

Laboratory investigations revealed haemoglobin level of 11.6 g/dl. White blood cell count, platelets, prothrombin time and partial thromboplastin time were within the normal range. The patient was consented for emergency laparotomy and taken to the operating room. Intraoperatively, we found a 4 cm full thickness perforation of the posterior vaginal fornix (Figure 2). The cervix was closed and there were no additional injuries identified. The herniated intra-abdominal contents were reduced and inspected. The bowel was edematous but viable and no injuries requiring intervention were identified. The edges of the vaginal perforation were debrided and the defect was closed in a continuous, full-thickness layer with 2–0 chromic catgut. Warm saline solution was then used to irrigate the peritoneal cavity. Post-operatively the patient was started on parenteral antibiotics. The patient had a prompt return of bowel habits, was quickly advanced to a full diet and was discharged on post-operative three. On post-operative day 10 the patient was seen in the out patient clinic. The patient was in good health and the midline incision was clean and dry. She was advised to refrain from sexual intercourse for eight weeks in order to allow the operative site to heal completely.

## Discussion

It is not uncommon to find lacerations of the posterior fornix. Several cases of posterior fornix laceration following sexual intercourse have been reported in the literature [1-3]. The majority of these cases were not full thickness and did not require laparotomy [4]. However, even without frank herniation of intra-abdominal contents, massive bleeding, shock, haemoperitoneum and peritonitis are reported [1-3]. Hall et al. and Tabriskey et al. reported cases of posterior fornix perforation with bowel evisceration [5,6]. Horace et al. reported a case of posterior perforation with peritonitis and haemoperitoneum [1]. Our patient presented with post-coital posterior fornix perforation with evisceration of intra-abdominal contents.

Trauma to the posterior fornix can occur as a result of direct injury during intercourse [7]. The posterior fornix is more vulnerable to injury as a result of a weaker layer of endopelvic fascia [4,8-11]. During coitus, the lower third of the vaginal wall contracts while the upper part expands and lengthens [12]. This places the endopelvic fascia of the posterior fornix under tension, which predisposes to injury. During forceful penile-vaginal intercourse there is an increase in the pressure within the vagina [4,8-11]. If the resultant injury violates the peritoneum, intra-abdominal contents may herniate into the vaginal cannel.

Vaginal evisceration is an emergency condition that needs prompt and meticulous intervention. Delay in management may result in complications such as shock, peritonitis, intestinal obstructions and hollow organ perforation [13]. Physicians should be aware of a misleading history when taken in the presence of family or partners. A delay in seeking medical care is common as patients try to avoid the perceived stigma of the injury. Sterile speculum and rectal examination are very important to establish the diagnosis. Midline laparotomy is the best approach to repair significant vaginal perforations when there is associated bowel evisceration though transvaginal and laparoscopy can be done in some selected cases.

## Conclusion

A high degree of suspicion for posterior fornix perforation is important in women with a history of recent sexual intercourse presenting with lower abdominal pain and vaginal bleeding. A thorough history and physical in the absence of family or partners is important to avoid unnecessary delays in diagnosis and treatment.

### Ethical approval

Written informed consent was obtained from the patient for publication of this report and accompanying images. A copy of the written consent is available for review by the Editor of this journal on request. Approval was also obtained from Research Ethics Board at the University of Dodoma and Iringa Regional Hospital.
